# Protocol: Precision engineering of plant gene loci by homologous recombination cloning in *Escherichia coli*

**DOI:** 10.1186/1746-4811-1-6

**Published:** 2005-09-29

**Authors:** Laura C Roden, Berthold Göttgens, Effie S Mutasa-Göttgens

**Affiliations:** 1 Broom's Barn Research Station, Higham, Bury St Edmunds, Suffolk IP28 6NP, UK; 2 Cambridge Institute for Medical Research, Hills Road, Cambridge, CB2 2XY, UK; 3 Dept. Mol. & Cell Biol., UCT, Private Bag Rondebosch, 7701, Cape Town, South Africa

## Abstract

Plant genome sequence data now provide opportunities to conduct molecular genetic studies at the level of the whole gene locus and above. Such studies will be greatly facilitated by adopting and developing further the new generation of genetic engineering tools, based on homologous recombination cloning in *Escherichia coli*, which are free from the constraints imposed by the availability of suitably positioned restriction sites. Here we describe the basis for homologous recombination cloning in *E. coli*, the available tools and resources, together with a protocol for long range cloning and manipulation of an *Arabidopsis thaliana *gene locus, to create constructs co-ordinately driven by locus-specific regulatory elements.

## Introduction

Plant bacterial artificial chromosome (BAC) resources are being generated for ever increasing numbers of species, providing scientists with long-range physical maps and associated sequence data for both model and crop plants. This provides opportunities for reverse genetics and functional studies at the level of the gene locus and above. The latter requires methods for the cloning and manipulation of large DNA fragments, without the limitations imposed by the need for suitably positioned restriction enzyme sites. Significant advances in this respect arose from the development of homologous recombination (HR) cloning in *Escherichia coli*, based on RecE/RecT (ET) [1,2] and λ RED operon gene products [3,4]. Essentially, in ET-based strategies, PCR-amplified linear DNA fragments with short regions of homology (~50 bp to 60 bp) are precisely targeted into any DNA sequence including high copy number plasmids, the *E. coli *chromosome and BACs. RED-based protocols rely on a defective λ prophage to provide functions that protect and recombine the linear DNA fragments, under the control of a temperature sensitive λ cl-repressor, with recombinogenic functions switched on at 42°C and off at 32°C. This fixed induction window helps to reduce unwanted rearrangements, allowing DNA to be stably cloned.

**Figure 1:**
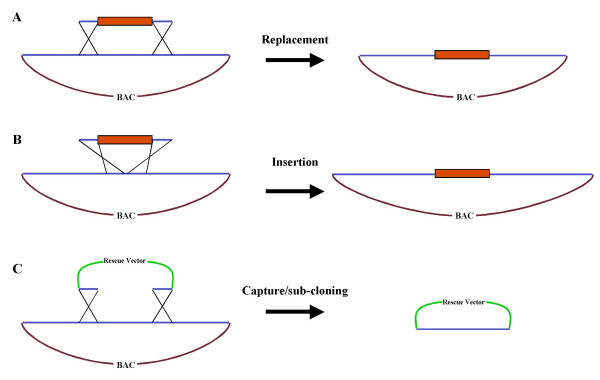
**Schematic representation of the basic applications of homologous recombination cloning in *E. coli *for genetic engineering**. Homologous recombination cloning in *E. coli *can be used for gene replacement (A), insertion (B) or sub-cloning of target sequences into alternative plasmid vectors. The recombination is mediated by linear DNA fragments (usually generated by PCR), including target site-specific homology arms and a counter selectable antibiotic resistance gene marker.

HR-cloning in *E. coli *is widely used in the biomedical research field and is becoming an established tool for BAC engineering in functional genomic studies [5]. Its applications include recombinogenic targeting for gene disruption or replacement and subcloning of BAC DNA by direct isolation of specific genomic regions. A general schematic of HR cloning is given in Fig. 1. Thus, the construction of transgenes for plant functional genomics or the next generation of genetically modified crop plants may benefit from the level of precision engineering offered by HR-cloning.

Our interest in long-range HR cloning was driven by a desire to create plant-specific tools and transgene constructs that target expression to the shoot apical meristem. We wanted to express the bean (*Phaseolus coccineus*) *GAPc2ox1 *(encoding GA 2-OXIDASE 1, which degrades bioactive gibberellin) in the shoot apex of sugar beet (*Beta vulgaris*) plants and study the effect on flowering. We present details of our constructs and the molecular tools (plasmids) developed to create these constructs by RED cloning.

## Materials

### Reagents

• *E. coli *strain EL250 (genotype DH10B [λ*cI857(cro-bioA)<> ara*C-P_BAD_*flpe*] where <> indicates that *cro-bioA *has been substituted with *ara*C-P_BAD_*flpe*) available from the authors of [3] who have developed a number of different strains including EL350 (with inducible araC-P_BAD_*cre*). These strains carry a defective λ prophage with *red *and *gam *recombination genes under the control of the λP_L _promoter and *exo *and *bet *tightly controlled by the temperature sensitive cI857 repressor. Exo and Beta provide recombinogenic function while Gam inhibits the *E. coli *RecBCD nuclease from degrading electroprated linear DNA fragments. The promoter of the *ara*BAD operon (P_BAD_) is induced by L-arabinose for *flpe *and cre expression enabling removal of sequences between *FRT *and *Lox*P sites respectively. We used EL250 to enable removal of the kanamycin gene in our FRT-mPGK-Tn5-neo-FRT cassette. **OUR RESULTS**: The marker gene was removed as described [3] and worked with 90%–100% efficiency and we were able to recover 100 s of colonies which had become kanamycin sensitive.

• Luria Bertani (LB) broth and plates supplemented with antibiotics as required

• Fully sequenced BAC, PAC or other clones with desired gene locus. Plant BAC and PAC clones are widely available from a number of different sources, including individual labs and organisations e.g. The Arabidopsis Biological Resource Centre (ABRC)  or the Nottingham Arabidopsis Stock Centre (NASC) at  and,  for rice genes and others. The *AtSTM *locus used in our experiments is cloned in BAC F24o1, sourced from the Arabidopsis Biological Resource Centre, Columbus, Ohio.

• pUC-based vectors to be used for making (i) the locus rescue gap-repair construct (must be counter selectable to the BAC/PAC), and (ii) the gene of interest (GOI) targeting cassette construct (must contain a counter selectable marker to the gap-repair construct).

• High fidelity *Taq *DNA polymerase. Preferably one which retains A-tails for TA cloning, e.g. the Expand High Fidelity PCR system (Roche Diagnostics).

• PCR primers – four locus-specific primers to amplify DNA fragments at the locus border flanks for the gap-repair rescue construct and two target site-specific primers (minimum 70 bp long) to generate GOI targeting products with destination site specific 5' and 3' homology arms.

• PCR product and gel purification kits e.g. the Qiagen QIAquick™ range and *Dpn*I restriction enzyme – used to remove plasmid templates from PCR reactions because it only cleaves methylated sites.

• General reagents for standard gene cloning and gel electrophoresis

### Equipment

• Orbital shaking incubator

• Orbital shaking water bath e.g. Grant OLS 200 – essential for induction of recombination functions in bacterial cells.

• Electroporator e.g. Bio-Rad *E. coli *Pulser

• PCR Machine

• Long wave UV transilluminator – long wave ultra violet light is less damaging to DNA during excision of bands from gels. UV-damaged DNA will not recombine efficiently.

• Electrophoresis equipment capable of field inversion gel electrophoresis (FIGE) or pulsed field gel electrophoresis (PFGE) e.g. BioRad CHEF DR-II, DR-III or Mapper™ XA, for efficient resolution of large DNA fragments

• Spectrophotometer for cell density quantification

• Temperature controlled centrifuge able to run at 4°C

## Protocol

The protocols outlined below describe the development of (i) an *AtSTM*-locus specific gap-repair rescue vector, (ii) a plant gene targeting construct with a removable kanamycin resistance marker cassette from pGK-FRT [6], under the control of both the bacterial *Tn5 *promoter and the mouse phosphoglycerate kinase (mPGK) promoter for selection in prokaryotes and eukaryotes respectively. This provides templates for PCR amplification of selectable gene fragments that can be precisely targeted into any desired gene locus; and (iii) a bean (*Phaseolus coccineus*) *GAPc2ox1 *transformation construct, co-ordinately driven by "all" *AtSTM *locus elements, designated p*STM*17::*GAPc2ox1*. We have also constructed a pENTR4-based *AtSTM *gap-repair rescue vector for the production of a Gateway™ (Invitrogen) compatible entry clone and generic T-DNA transformation constructs as well as an *mgfp5-ER *targeting cassette. The p*STM*17::*GAPc2ox1 *was successfully transformed into sugar beet, demonstrating for the first time that the mouse PGK promoter is fully functional in transgenic plants, thus enabling the direct exploitation of existing mammalian tools.

### Key steps in the EL250 RED-HR locus rescue and engineering procedure

1. Design of PCR primers for amplification of locus rescue (retrieval) homology arms and also for GOI targeting

2. Construction of a gap-repair locus rescue vector.

3. Construction of a targeting vector containing the GOI upstream of a counter selectable marker (different from that in the gap-repair construct).

4. Electroporation of EL250 cells with the BAC or clone containing the desired gene locus and preparation of electrocompetent BAC/EL250 cells induced for Exo, Beta and Gam functions.

5. Performance of gap-repair locus rescue, in cells treated as above; selection of recombinants and confirmation by restriction digestion analysis and sequencing. Transformation of the rescued locus plasmid into fresh EL250 cells.

6. PCR amplification, purification and quantification of the GOI targeting cassette and its site-specific recombination into the rescued locus plasmid in EL250 cells. Selection and confirmation of recombinants as above.

The recombineered plasmid is now ready for application in functional analyses as desired.

### Primer design and plasmid constructs

#### Primers

**Figure 2:**
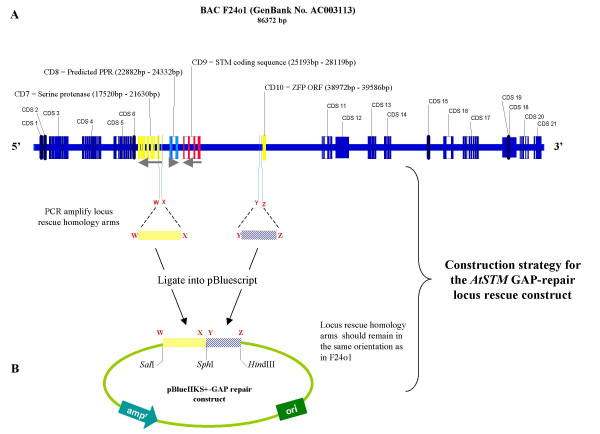
**Maps of the *Arabidopsis *BAC F24o1 and the *AtSTM *gap-repair construct**. A: A physical map of BAC F24o1, showing the relative positions of the 21 coding sequences (CDS), including *STM *(CDS 9) and its immediate neighbours (CDS 7, 8 and 10) which, are illustrated in different colours and greater detail to show the exon blocks making up each open reading frame. Grey arrows show the orientations of the serine protease, predicted pentatricopeptide repeat protein (PPR), *SHOOTMERISTEMLESS (STM) *and Zinc Finger Protein (ZFP) open reading frames. B: The gap-repair construct and a schematic representation of the basic protocol used to generate the *AtSTM *downstream (yellow vertical dashed line: W - X) and upstream (blue diagonal dashed line: Y - Z) homology arms to create it in the pBluescriptII KS+ vector backbone. W, X, Y and Z are the PCR primers used to generate each homology arm fragment The *Sal*I, *Sph*I, and *Hind*III cloning sites were incorporated into the PCR primers, the sequences of which are given in Additional file 1.

Primer sequences for the *AtSTM *HR rescue protocol described here are given in Additional file 1. Careful attention must be paid to the design of primers for generating locus rescue (LR) homology arms (HA) to ensure that their orientation in the resultant gap-repair vector is correct for DNA double stranded break repair homologus recombination. A total of four short (18 to 20 bp) primers will be required and can if necessary, include restriction sites to enable cloning into the gap-repair vector so that the gap-repair construct can be linearised between the LR-HAs. Fig. 2A shows our *AtSTM *gap-repair construct.

For the GOI targeting cassette, primers must include at least 30 to 50 bp at the 5' end, to provide homology arms for site-specific recombination. The target site sequence must not have any mismatches as this will inhibit recombination. It is therefore essential to source primers from suppliers able to guarantee sequences of long primers. Our primers were custom made by Sigma Genosys.

#### Plasmid constructs

**IN OUR HANDS**: Creation of these basic plasmids was the key limiting step as it is dependent on conventional cloning and therefore, on the availability of suitably placed restriction sites. However, once constructed, the gene targeting constructs can be used to target the expression cassette into any desired site whereas the gap-repair construct is suitable only for subcloning the specific gene fragment/locus. Our targeting construct backbone has therefore been designed to include a plant-specific polyA signal (Nopaline synthase (nos) termination sequence), for generic use with any plant cDNA sequence.

##### *AtSTM *gap-repair construct

Using BAC F24o1 DNA template (represented in Fig. 2A), and the Expand High Fidelity DNA polymerase PCR system (Roche Diagnostics) we amplified 564 bp (incorporating 5'*Sal*I and 3'*Sph*I sites) and 479 bp (incorporating 5'*Sph*I and 3'*Hind*III sites) homology arms respectively at the downstream and upstream flanks of the *AtSTM *locus (Fig. 2B). At the start of our project, the pentatricopeptide repeat protein (PPR) downstream of the STM coding sequence was annotated as a predicted ORF and we therefore opted to include it in the STM locus fragment. Now, it would be excluded as the locus boundary. PCR reactions included primers *Hind*III 3' hyp/*Sph*I 5' hyp or Sprot H1 *Sal*I/Sprot H1 *Sph*I at 0.3 μM each and were incubated for 1 cycle at 94°C for 2 min. followed by 30 cycles of 94°C for 15 sec; 64°C for 30 sec; 72°C for 1.5 min; and 1 cycle of 72°C for 5 min. The individual products were then sub-cloned into pGEM-T Easy (Promega), recovered and cloned into pBluescript II SK+ (Stratagene) in a three-way ligation reaction, to create the gap-repair construct.

**NOTE***: In our hands, cloning of PCR products is more efficient if we shuttle them via a PCR cloning vector. Any TA cloning vector is suitable. In this case, it is important to ensure that the proof reading activity of the Taq Polymerase used does not remove A-tails*.

##### Targeting construct backbone

**Figure 3:**
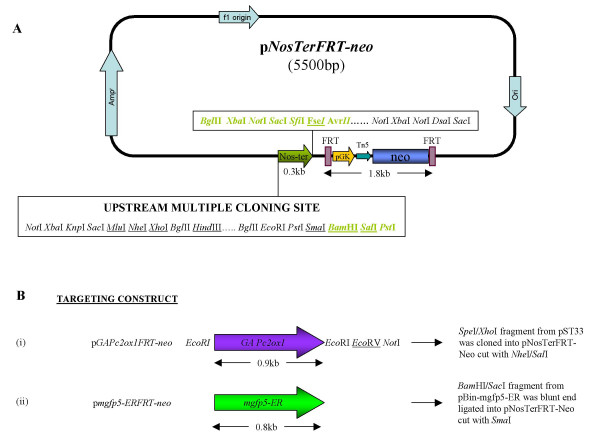
**Map of the targeting plasmid backbone p*NosTerFRT-neo *and *GAPc2ox1/mgfp5-ER *targeting cassettes**. A: Structure of the targeting plasmid backbone pNosTerFRT-neo, showing the relative positions of the Nopaline synthase polyA signal sequence (NosTer), the FRT-mPGK-Tn5-neo-FRT selection cassette and the restriction sites mapped up and downstream of the NosTer sequence. Upstream sites may be used for cloning any GOI cDNAs to create targeting constructs. Sites shown in green were introduced with the NosTer fragment; all other sites originate from PolyGFP3. Unique sites are underlined. B: Representations of GOI sequences with available upstream and downstream restriction sites as appropriate, together with the cloning strategies employed to clone them into pNosTerFRT-neo to make the targeting cassette constructs.

The 1.8 kb *FRT-mPGK-Tn5-neo-FRT *cassette was PCR amplified with *Sac*II in the 5' primer (PGK-FRT upper) and *Apa*I in the 3' primer (PGK-FRT lower) from pPGK-FRT (obtained under a Material Transfer Agreement from Dr Francis Stewart, EMBL, Heidelberg – now at University of Technology, Dresden) and cloned into *Sac*II/*Apa*I cloning sites downstream of the Green Fluorescent Protein (*GFP*) gene of polyGFP3 (a kind gift from Dr E. Amaya, Gurdon Institute, Cambridge). The *GFP *gene was then replaced with the Nopaline synthase Terminator (NosTer) from pAL69 (pFC6 with NosTer in the multiple cloning site. A kind gift from Dr Dave Lonesdale at the John Innes Centre, Norwich, UK), to create the basic plant gene targeting vector pNosTerFRT-neo (Fig. 3A), which can be used to receive any GOI.

##### *GAPc2ox *targeting construct

The full length *GAPc2ox1 *cDNA in plasmid pST33 (a kind gift from Drs Andy Philips and Peter Hedden, Rothamsted Research) was excised with *Spe*I/*Xho*I and cloned into the *Nhe*I/*Sal*I site of pNosTerFRT-neo (Fig. 3Bi).

##### *mgfp5-ER *targeting cassette

The *mgfp5-ER *gene (GenBank U87974) was isolated from pBIN35S-mgfp5-ER (a kind gift from Dr Jim Haseloff, Department of Plant Sciences, University of Cambridge, UK) as a *Bam*HI/*Sac*I fragment and cloned into the *Sma*I site of pNosTerFRT-neo (Fig. 3Bii)

**WARNING! ***GUS *reporter cassettes are not suitable as they are able to recombine with the endogenous (chromosomal) *E. coli *gene during HR cloning.

### RED Cloning Protocol

Original methods and information on how to obtain host cells can be found at the recombineering website 

#### A: Preparation of electrocompetent EL250 cells

These cells will be periodically used to receive new plasmids as they are constructed and required for recombineering. It is therefore advisable to make and store a sizable batch.

1. Streak out cells on LB plates and grow at 32°C. The cells are temperature sensitive and will die at 37°C

2. Inoculate a single colony into 5 ml LB and grow overnight.

3. Inoculate 1 ml of the overnight culture into 50 ml LB in a 500 ml flask and grow at 32°C with shaking at 200 revolutions per minute (rpm) until the cells density has reached OD_600 _= 0.5 – 0.8.

4. Spin cells at 4°C (rotor must be pre-cooled) and wash with 5 ml ice cold sterile distilled water. Spin and discard supernatant. Repeat wash with 5 ml aliquots of ice cold SDW two more times.

5. Finally resuspend cells in 500 μl of ice cold sterile distilled water and aliquot 100 μl lots into cooled 1.5 ml microfuge tubes.

**NOTE***: Cells can be used immediately or re-suspended in ice cold sterile 10% (v/v) glycerol and stored at -80°C until required*.

#### B: Transformation of BAC F24o1 and induction of recombinogenic function in EL250

1. On ice, add 10 ng-100 ng of F24o1 DNA to 50 μl of competent EL250 cells. Mix by gentle pipetting and transfer to a pre-cooled 0.1 cm electroporation cuvette.

2. Pulse at 1.75 kV in a Bio-Rad E. coli Gene Pulser. Immediately add 1 ml LB broth and incubate at 32°C for 1–1.5 h in a shaking incubator set at 200 rpm

3. Plate cells on LB kanamycin and select for transformants. **NOTE**: *It is advisable at this stage to check the integrity of the BAC clone by restriction digestion analysis, to ensure that there have been no rearrangements*.

4. Grow F24o1/EL250 cells as described in A: steps 1 – 3 except that all LB media must be supplemented with kanamycin (or relevant antibiotic) to select for the BAC. **NOTE**: *before the next step, ensure that the shaking water bath is switched on early and stabilised at 42°C ready for use and pre-warm the conical flask. An ice slurry bath must also be made ready – DO NOT USE JUST ICE – it will not cool cells fast enough*.

5. For induction, transfer 10 ml of the growing culture into a pre-warmed 250 ml conical flask and incubate in the water bath at 42 °C with shaking at 200 rpm for a total of 15 min. **NOTE***: Keep the remaining 40 ml of culture at 32 °C to act as a non-induced control*.

6. Immediately after 15 min. place the flask in the ice slurry bath and swirl by hand to quickly cool down the cells. Include a similar flask with 10 ml of non-induced control cells – this will be cooled down and treated in the same way as the test cells from now on. **NOTE**: (i) *Induced cells must be used immediately as they will lose activity above 0°C. Therefore it is important to work quickly from now on. However, cells may be kept on ice for a total of 40 min. without significant loss of activity. (ii) Ensure that the centrifuge and rotor are pre-cooled to 4 °C before the next step*.

7. Centrifuge the 10 ml aliquots of induced and control cells for 8 min at 5500 *g *and at 4 °C. Retrieve pellets and wash three times in 1 ml ice cold sterile distilled water and centrifuge as above. **NOTE**: *To save time, washing steps can be carried out in 1.5 ml microfuge tubes keeping everything ice cold and centrifuging at 4 °C for 20 seconds each time*.

8. After final wash, re-suspend the cell pellet in 100 μl of ice cold sterile distilled water. This is enough for two electroporation transformation reactions.

#### C: AtSTM locus rescue from BAC F24o1 by gap-repair HR

**Before starting**: Ensure that purified and linearised gap-repair vector is available at concentrations suitable to deliver 10 to 100 ng in volumes up to 10 μl. We strongly recommend gel quantification with known standards as we find this more accurate than OD_260 nm _measurements.

**N.B. **All HR experiments should be carried out with the induced and un-induced control cells in parallel.

**Figure 4:**
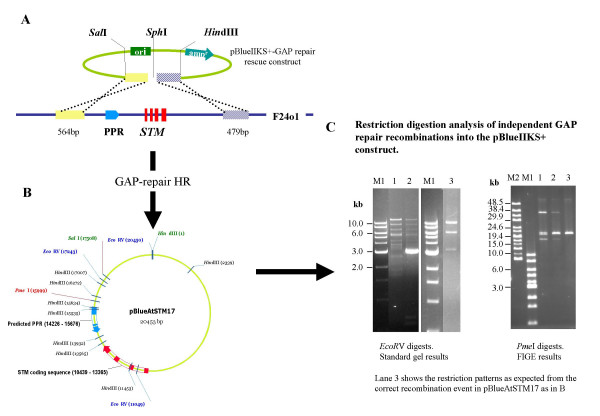
**gap repair HR rescue of the *AtSTM*locus**. Schematic representation of the HR rescue of the *AtSTM *locus into the pBluescript gap repair vector (A) and the resultant pBlueAtSTM17 construct as expected from the correct recombination event. Diagnostic *Eco*RV sites are shown in blue, *Pme*I site in red, together with their co-ordinates within pBlueAtSTM17. *Sal*I and *Hind*III sites originally engineered in the homology arms of the gap repair vector are shown in green. (B). The results of *Eco*RV and *Pme*I restriction digestion of three plasmids resulting from independent recombination events from a single experiment, showing that only the plasmid in lane 3 resulted from the correct recombination. M1 = 1 kb ladder (New England Biolabs); M2 = High Molecular Weight Marker (Invitrogen).

1. Using linearised gap-repair construct DNA, electroporate induced competent F24o1/EL250 cells as described in B: steps 1 – 2.

2. Select recombinants on LB supplemented with antibiotic marker for the gap-repair vector. We used pBluescriptII KS+ and therefore selected on LB ampicillin. The use of pBluescript also limits the size of insert which can be rescued and 17 kb was the largest fragment we were able to retrieve by gap repair HR cloning.

3. Recover recombinant plasmids and confirm correct recombination events by restriction digestion analysis and sequencing. This is important since incorrect events may still be selectable with the antibiotic marker. **OUR RESULTS**: The number of colonies recovered was typically small (2 – 4) but of these, 50% were correct. The remainder were the result of illegitimate recombination events. See Fig. 4 for our HR strategy and the result of our *AtSTM *gap-repair rescue to give the plasmid pBlueAtSTM17. **NOTE**: *Because of the large DNA fragments involved, it is advisable to use Field Inversion Gel Electrophoresis or Pulsed Field Gel Electrophoresis as appropriate for clarity in resolution*.

**TROUBLE SHOOTING**: Growth of un-induced colonies on selective plates suggests incomplete digestion of the gap-repair construct during linearization. However, the number of colonies should be low (we typically recovered 5 – 10 colonies from un-induced cells). Otherwise repeat with improved digestion and/or gel purification of the linearised gap repair construct.

4. Transform the rescued plasmid into fresh EL250 cells and prepare induced competent cells as described, ready for the locus targeting experiment. We designated these cells STM17/EL250 because they contained rescued ~17.5 kb of the *AtSTM *locus. **NOTE**: *For our application, we use a protoplast based direct transformation method and therefore opted to use pBluescript as the backbone for our gap repair and eventual transformation construct. However, for Agrobacterium-based systems, we recommend using a Gateway compatible Entry vector (available from Invtrogen: ) as this will enable subsequent transfer of the captured, manipulated locus into a T-DNA binary destination vector for example the ones available from Plant Systems Biology *(*VIB-Gent University: *) *or the pEarlyGates vectors, details of which can be found at the website: *.

Recently, we have successfully created an *AtSTM *locus rescue vector based on the Invitrogen pENTR4 Gateway™ compatible vector in which we plan to capture/manipulate the locus as described and determine the success rate of transfer into a promoterless T-DNA destination vector pB7WG2Δ35S (based on pB7WG2 from VIB-Gent University). These are newly available resources that should enable the creation of constructs for the more generic *Agrobacterium*-mediated plant transformation systems.

**WARNING!**: Direct use of T-DNA vectors as gap-repair constructs in RED cloning although attractive, may prove problematic because of the common use of a limited number of identical or very similar promoter and polyA signal sequences, which if also present in the targeting cassette will result in illegitimate recombination events. For this reason, we did not attempt any experiments with T-DNA vectors, opting instead to go via the Gateway™ system as detailed above.

### D: Replacement of AtSTM exon1 by in-frame fusion of the promotorless GAPc2ox-FRT-neo-FRT targeting cassette

**Before starting**: The purified, *Dpn*I treated and quantified PCR amplified GOI targeting cassette should be made ready for this experiment.

**Figure 5:**
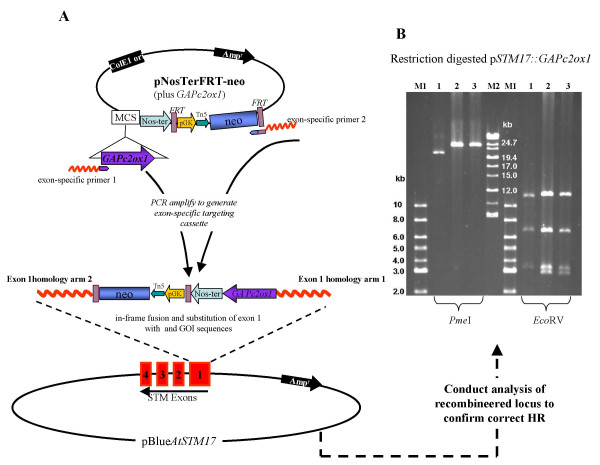
**Replacement of *AtSTM *exon 1 by in-frame fusion of *GAPc2ox1***. A: Schematic representation of the strategy used to generate the *GAPc2ox1 *cassette from pNosTerFRT-neo (plus *GAPc2ox1*) plasmid and target it into the *AtSTM *locus (captured in pBlueAtSTM17) by in-frame substitution of exon 1 sequences. B: FIGE gel results of *Pme*I and *EcoR*V digested p*STM17::GAPc2ox1 *from two independent recombination events (lanes 2 and 3), clearly showing the increased size of the linearised construct (*Pme*I digest) and the additional fragment (*Eco*RV digest) due to the recombination of the *GAPc2ox1 *cassette. Lane 1 shows the control result with the original pBlue*AtSTM17*. M1 = 1 kb ladder (New Endgland Biolabs); M2 = High Molecular Weight Marker (Invitrogen).

1. Using up to 100 ng of the PCR amplified *GAPc2ox1-FRT-neo-FRT *targeting cassette, electroporate induced STM17/EL250 cells as described in B: steps 1 – 2. The targeting cassette was amplified with HotStar Taq DNA polymerase (Qiagen) and primers 28001 frtlow and 2oxexon1 (0.3 μM each) in a 50 μl reaction volume. Incubation conditions were 1 cycle 95°C for 1 min followed by 20 cycles of 94°C for 15 sec; 68°C for 4.5 min. (with a 5 sec. time increment in each cycle); followed by 1 cycle of 68°C for 10 min.

2. Select recombinants on LB kanamycin. Recover plasmids and confirm by restriction digestion analysis and sequencing. Plasmids are now ready for application in functional assays. **OUR RESULTS**: We recovered many colonies at this stage (100 s), of which ~6 % were correct by restriction digestion analysis and sequencing. For example, we typically screened between 30 and 35 colonies from which two were correct. See Fig. 5 for the results of our GOI targeting experiment. **NOTE**: *It is advisable at this stage to at least sequence across the recombination site into the GOI cassette to confirm the integrity of the gene cassette before proceeding with functional assays in transgenic plants*.

**TROUBLE SHOOTING**: High un-induced colony numbers on selective plates suggest targeting cassette template contamination instead of recombination. Check *Dpn*I digests and use this in combination with gel purification to remove template DNA from the target cassette PCR product prior to electoporating for HR. In our experience, if the number of colonies from un-induced cells is at least 50 – 100 fold less than from the induced cells, then it was worth screening colonies from induced cells.

**Figure 6:**
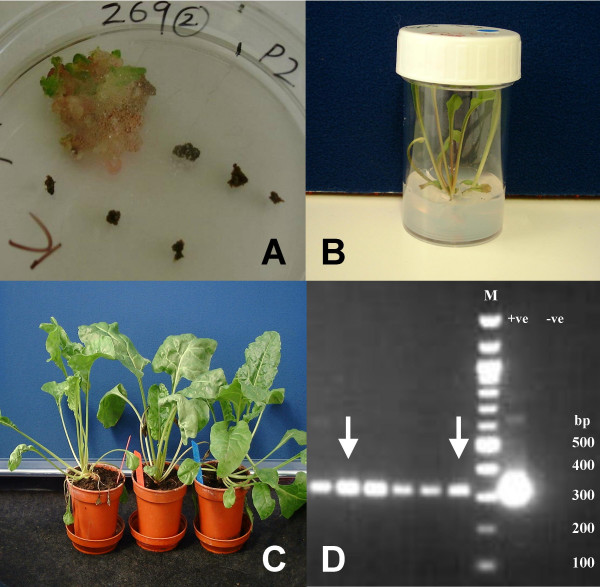
**Kanamycin selection and regeneration of transgenic sugar beet guard cells transformed with the *pSTM17::GAPc2ox1 *constructs containing the neomycin gene driven by the mouse PGK promoter**. A: Transgenic sugar beet callus grown from transformed sugar beet guard cell protoplasts cultured on medium supplemented with kanamycin at 100 μg ml^-1^, clearly showing a difference between the healthy (green) callus and the dead (brown) non-transgenic callus. B: Shoots regenerated from transgenic sugar beet callus as above. C: Resultant transgenic seedlings in compost. D: Agarose gel showing the results of neomycin gene-specific PCR conducted on genomic DNA template extracted from sugar beets generated from two independent transformation events with *pSTM17::GAPc2ox1 *including the kanamycin driven by the mouse PGK promoter (arrows) compared with individuals from lines transformed with constructs containing a kanamycin cassette driven the CaMV35S promoter (the remaining PCR product bands). Sequences from other regions of the *pSTM17::GAPc2ox1 *construct were also detected by PCR and Southern blot hybridisation (results not shown). +ve = DNA plasmid template control, -ve = DNA template-free control; M = New England Biolabs 100 bp ladder. Diagnostic PCR for the neomycin gene sequences was performed using the primers Neo-For 5' CAG GAT GAT CTG GAC GAA GA 3' (Tm = 57.3 °C) and Neo-Rev 5' AAG AAG GCG ATA GAA GGC GA 3' (Tm = 57.3°C). The reactions (Qiagen Master Mix) contained 10 μM of each primer, and 50–100 ng genomic DNA template and were incubated at 94°C for 3 min, followed by 30 cycles of 94°C for 15 sec.; 55°C for 30 sec.; 72°C for 1 min and one cycle of 72 °C for 2 min.

Our final recombineered construct was designated *AtSTM17:: GAPc2ox1 *and was transformed into sugar beet guard cell protoplasts [7] from which we successfully selected transgenic callus and shoots under kanamycin selection driven by the mouse PGK promoter in the FRT-mPGK-Tn5-neo-FRT cassette (Fig. 6). We are now in the process of conducting phenotypic analyses of our *AtSTM17:: GAPc2ox1 *plants.

## Comments

Manipulation of large DNA fragments to make complex constructs for functional genomics or genetic engineering for crop improvement is possible using HR cloning in *E. coli*. We have successfully used HR cloning in *E. coli *to sub-clone the *Arabidopsis thaliana SHOOTMERISTEMLESS *(*STM*) gene locus from a BAC clone into pBluescript and to replace exon 1 sequences with a Gibberellin 2-oxidase cDNA gene-of-interest cassette tightly linked to an FRT-flanked kanamycin selection marker gene. This cassette is of generic use because firstly, it can be targeted/recombineered into any locus/destination site. Secondly, the kanamycin resistance gene is under the control of both the bacterial Tn5 promoter and the mouse phosphoglycerate kinase promoter (mPGK), which respectively allow for selection in prokaryotes and eukaryotes. We have now demonstrated the utility of the mPGK promoter for driving expression in transgenic plants and this suggests that there may well be increased scope for plant scientists to directly benefit from existing molecular genetic tools developed for application in the biomedical field.

*E. coli *ET- and RED-HR cloning are well established technologies within the biomedical field and they have many uses besides the creation of transformation constructs with long-range regulatory elements. The identification of regulatory elements or locus control regions located at a distance from the gene sequence can be assisted by this strategy. Point mutations, deletions or insertions, gene fusions and antisense constructs can be engineered on any BAC for functional genomics studies. The scope for plant science is further enhanced by the recently reported application of HR to convert BACs into binary vectors [8] together with (i) the availability of a BAC-based physical map of *A. thaliana*, (ii) freely available genome sequence information through the *Arabidopsis *Genome Initiative, (iii) access to rice sequence data and BAC resources through The Institute for Genomic Research (TIGR) and the Rice Genome Resource Center (RGP).

Resources for RED/ET cloning are available from Neal Copeland and Nancy Jenkins for both profit and non-profit organisations. Details can be found at the following website: . The commercial company GeneBridges  also offers reagents and a DNA engineering service.

## Abbreviations

BAC = Bacterial artificial chromosome; bp = base pairs; FIGE = field inversion gel electrophoresis; GA 2ox = gibberellin 2-oxidase; GFP = gree fluorescent protein; GOI = gene of interest; HA = homology arm(s); HR = homologous recombination; LB = Luria Bertani medium; LR = Locus rescue; mPGK = mouse phosphoglycerate kinase promoter; OD = optical density; PCR = polymerase chain reaction; ORF = open reading frame; rpm = revolutions per minute, UV = ultraviolet.

## Competing interests

The author(s) declare that they have no competing interests.

## Authors' contributions

ESM-G and BG conceived of the study and participated in its design. ESM-G and LCR drafted the manuscript. LCR participated in the design of the study and carried out most of the experimental. ESM-G directed the work, participated in experimental work. All authors read and approved the final manuscript.

## Supplementary Material

Additional File 1 **PCR Primer Sequences**. Details of the primers used isolate and manipulate the *AtSTM *gene locus by homologous recombination in *E. coli *EL250 Click here for file
