# Supplementary material for: Protocol: Precision engineering of plant gene loci by homologous recombination cloning in Escherichia coli
Source: Plant Methods. 2005 Sep 29;1:6. doi: 10.1186/1746-4811-1-6 (PMC1277019; doi:10.1186/1746-4811-1-6)
Supplement: Additional File 1 — PCR Primer Sequences. Details of the primers used isolate and manipulate the AtSTM gene locus by homologous recombination in E. coli EL250 [file 1746-4811-1-6-S1.doc]

# PRIMER SEQUENCES

## *STM* locus-specific nucleotides are shown in red, GOI specific nucleotides in black, incorporated restriction sites in bolt italics, and nucleotides to protect the restriction site are in bold

| **Name** | **Sequence** | **Tm** |
| --- | --- | --- |
| Sprot H1 *Sal*I | 5’ **AGCT*GTCGAC***CAGACTTGTTGAGGAAGTTCCA 3’ - primer W | 69.5 ˚C |
| Sprot H1 *Sph*I | 5’ **AGCT*GCATGC***CAAAGCTATGGCGTTAGAAGCA 3’ - primer X | 69.5 ˚C |
| *Sph*I 5’ hyp | 5’ **AGCT*GCATGC***TTGTATCGGATCCGAAACTA 3’ - primer Y | 66.8 ˚C |
| *Hind*III 3’ hyp | 5’ **AGCT*AAGCTT***GTCCGTTAGGGAAGACATCA 3’ - primer Z | 66.8 ˚C |
| PGK-FRT upper | 5’ CCTATGCTACTCCGTCG 3’ | 56.7 ˚C |
| PGK-FRT lower | 5’ TCCCGGCGGATTTGTCCTACTCAGGAGAGCG 3’ | 82.5 ˚C |
| 28001 frtlow | 5’TCATAGGACACATCGGACCATCACTATTATCCCCGGCAAAAGCCATTGGACGGATTTGTCCTACTCAGGAGAGCG 3’ | 92.3 ˚C |
| 2oxexon1 | 5’TATAGCAAAGCCAAAGTGAATAATAATACTAGTGAGAGAAAGAGAAG**ATG**GTTGTTCTGTCTCAGCCAGC 3’ | 82.4 ˚C |
